# Chain mediation of resourcefulness and self-perceived burden between coping styles and psychological distress in stroke patients: a cross-sectional study

**DOI:** 10.3389/fpsyg.2025.1560348

**Published:** 2025-09-05

**Authors:** Shuang Liu, XianYi Huang, Jie Yue, Yan Liu, Yu Li, Li Chen

**Affiliations:** ^1^Department of Oncology, Affiliated Hospital of Southwest Medical University, Luzhou, China; ^2^School of Nursing, Southwest Medical University, Luzhou, China

**Keywords:** stroke, psychological distress, coping style, resourcefulness, self-perceived burden

## Abstract

**Objective:**

To explore the chain mediating roles of resourcefulness and self-perceived burden in the relationship between coping styles and psychological distress in stroke patients.

**Methods:**

This study is a cross-sectional investigation conducted from April to December 2023. A convenience sample of hospitalized stroke patients (*n* = 432) was obtained in Sichuan Province, China. A demographic questionnaire, Medical Coping Mode Questionnaire, Resourcefulness Scale, Self-Perceived Burden Scale and psychological distress Thermometer were used to conduct the survey. Mediation effect testing was conducted using SPSS 25.0.

**Results:**

Coping style, resourcefulness, self-perceived burden and psychological distress were significantly related to each other (*p* < 0.05). The chain mediation effects of resourcefulness and self-perceived burden between coping styles (confrontation and avoidance) and distress were significant.

**Conclusion:**

Resourcefulness and self-perceived burden mediated the relationship between coping styles (confrontation, avoidance) and psychological distress. The results imply that intervention from the perspective of coping style, resourcefulness and self-perceived burden may help to alleviate psychological distress in stroke patients.

## 1 Introduction

The incidence of stroke remains high globally and is a major cause of death and disability (GBD 2019 Stroke Collaborators, 2021; GBD 2021 Diseases and Injuries Collaborators, 2024). Experts predict that stroke will become the second leading cause of global disease burden by 2050 (GBD 2021 Forecasting Collaborators, 2024). Stroke has a sudden onset and rapid progression and may lead to various post-stroke complications. Consequently, survivors often face multiple challenges such as economic, physiological, and psychological difficulties, which frequently result in significant distress. In the United States, 11.3% of adult stroke patients suffer from severe psychological distress (Dong et al., 2022), while in China, 55.22 to 78.03% of patients experience significant psychological distress (Gu et al., 2021; Du, 2018), and 26.6% of patients experience suffer from severe psychological distress (Li, 2021). Psychological distress is a continuous psychological process. Mild distress is a normal emotional response, including worry and fear, but it can escalate to more severe forms such as anxiety and depression (Carney and Freedland, 2002). Severe psychological distress harms patients’ mental health and affects physiological recovery in stroke patients (Huang et al., 2017) and their quality of life (Minshall et al., 2021), this has an adverse effect on rehabilitation. Moreover, greater psychological distress is associated with higher overall mortality rates (Hockey et al., 2022).

Psychological distress in stroke patients is associated with their self-perceived burden (SPB) which is a psychological response where individuals experience worry, guilt, burden, and reduced self-esteem due to the negative impact their illness and care needs have on caregivers (McPherson et al., 2010). Neurological impairments caused by stroke often transform an otherwise self-sufficient individual into one with compromised abilities, which will cause individuals to doubt their abilities, and then generate guilt, self-reproach and other experience of Wei et al. (2020). Feelings of burden will deepen the experience of psychological distress (Hill and Frost, 2022).

Although the challenges of stroke may increase patients’ perceived burden and psychological distress, numerous studies show that positive coping styles and a high level of resourcefulness are beneficial for mental health. Efective coping can alleviate negative emotions or resolve problems (Li and Feng, 2017), thereby allowing patients to experience less perceived burden and psychological distress (Liu S., 2023; Liu et al., 2024). In contrast, negative coping may contribute to the development of anxiety and depression (Li et al., 2021). Resourcefulness is also closely related to mental health. Resourcefulness refers to the ability to independently, to manage daily tasks, as well as the capacity to seek external assistance when unable to function independently. It includes personal resourcefulness (also known as learned resourcefulness) and social resourcefulness (Zauszniewski et al., 2006). Individuals with higher levels of personal resourcefulness possess better internal coping resources, making it easier for them to achieve effective coping, which is crucial for maintaining and promoting their physical and mental health (Zauszniewski and Burant, 2020). Individuals with higher social resourcefulness are also more likely, actively, to seek support from family and the community, which can help alleviate their perceived burden and psychological distress (Bekhet, 2015).

**Figure 1:**
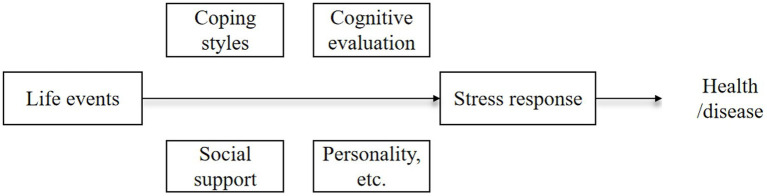
Stress system.

In summary, psychological distress is closely related to self-perceived burden, coping style, and resourcefulness. While existing research has demonstrated the pairwise relationships among these factors, the complex pathways connecting all four have yet to be elucidated. The stress system model (Jiang, 2014, 2006) (Figure 1) of stress suggests that the process from stressors to stress responses is influenced by various factors, and differences in stress responses can lead to different health outcomes. Similarly, resourcefulness theory emphasizes that an individual’s resourcefulness is shaped by background factors (such as illness) and, ultimately, affects their health outcomes (Zauszniewski, 2016). In this study, stroke serves as the stressor and psychological distress as the stress outcome, reflecting the individual’s mental health. Self-perceived burden is a psychological reaction of self-blame and guilt, which is part of the stress response, and both coping styles and resourcefulness may potentially influence the pathway from stressor to stress outcome. This leads to our research question: After suffering a stroke, how does the patient’s coping style further affect psychological distress through resourcefulness and self-perceived burden?

To explore this question, we hypothesized that coping styles influence psychological distress through their effects on resourcefulness and self-perceived burden in stroke patients. Accordingly, a cross-sectional survey was conducted involving stroke patients to preliminarily uncover the psychological mechanisms through which coping styles affect psychological distress.

## 2 Materials and methods

### 2.1 Study design and participants

This study is a cross-sectional survey. From April to December 2023, stroke patients hospitalized in four tertiary A hospitals in Sichuan Province, China were conveniently selected as the survey subjects. Inclusion criteria include: ① Patients who meet the diagnostic criteria and are diagnosed with stroke; ② Age ≥ 18 years with sufficient communication abilities; ③ Diagnosed with stroke for ≥ 7 days; ④ Conscious with stable vital signs, aware of their medical condition, and voluntarily participating in this study. Exclusion criteria included patients with cognitive impairments and severe organ failure (heart, liver, kidney, etc.), as well as those currently undergoing psychological therapy or cognitive-psychological interventions. According to Cohen’s sample size estimation method (Cohen, 2013), the sample size should be 10–15 times the number of observed variables. With 23 independent variables in this study and accounting for 20% invalid samples, the final sample size is estimated to be between 276 and 414 participants.

### 2.2 Procedures

Prior to the survey, approval was obtained from the participating institutions, and the study was reviewed and approved by the ethics committee (No. KY2023108). A pre-surveys was conducted with 30 patients to test the reliability of the scales and further refine the general information questionnaire. During the survey, investigators rigorously screened participants to ensure they complete the questionnaire in an undisturbed environment, free from interference or suggestion. After the survey, two investigators (LS & HXY) cross-checked the collected questionnaires on the same day. Any omissions or errors were immediately corrected or supplemented. To reduce bias in the data collection process, both interviewers underwent standardized training before the survey, following the same instructions and standard operating procedures. For patients who had difficulty understanding the questionnaire or had visual impairments, a one-on-one approach was adopted, where the investigator objectively stated the questions and options, then completed the responses on behalf of the patient. This study was conducted in accordance with the STROBE Statement (von Elm et al., 2007) (checklist in the Supplementary material).

### 2.3 Measurement

#### 2.3.1 General information questionnaire

The general information questionnaire was compiled by the researcher based on the relevant literature and the opinions of clinical experts and statistical experts. Involving nine variables of sociodemographic data (gender, age, residence, education level, occupation, marital status, average monthly household income, primary caregiver, medical payment method) and six variables related to disease (stroke type, first onset, timely treatment within 6 h of onset, comorbid chronic diseases, disease duration, and Activies of Daily Living (ADL) score).

#### 2.3.2 Medical coping mode questionnaire (MCMQ)

Developed by Feifel et al. (1987) and translated into Chinese by Shen and Jiang (2000), the questionnaire consists of 20 items divided into three dimensions: confrontation, avoidance, and resignation. It employs a Likert 4-point rating scale, with higher scores indicating a patient’s coping tendencies. The questionnaire has been widely used with good reliability and validity. In this study, the Cronbach’s alpha of the three dimensions of confrontation, resignation, and avoidance measured in stroke patients were 0.863, 0.780, and 0.926, respectively.

#### 2.3.3 Resourcefulness scale

The scale was compiled by Zauszniewski et al. (2006) and translated into Chinese by Ke et al. (2015). It consists of 28 items across two dimensions: personal resourcefulness and social resourcefulness, rated on a 6-point scale from 0 to 5, with a total score range of 0 to 140. Higher scores indicate higher levels of resourcefulness. Following translation, the Cronbach’s alpha for the scale was 0.825, test–retest reliability was 0.852, and the interdimensional correlation coefficient was 0.432, indicating good internal consistency (Ke et al., 2015). In this study, the Cronbach’s alpha of this scale in stroke patients was 0.844, and the Cronbach’s alpha of the personal and social resourcefulness were 0.872 and 0.710, respectively.

#### 2.3.4 Self-perceived burden scale (SPBS)

This scale was compiled by Cousineau et al. (2003) and translated into Chinese by Wu and Jiang (2010), the scale comprises 10 items rated on a 5-point Likert scale (1–5), with a total score ranging from 10 to 50. Higher scores indicate a greater self-perceived burden, with scores <20 suggesting no significant SPB, 20–29 indicating mild SPB, 30–39 indicates moderate SPB, and 40–50 indicates severe SPB. The Cronbach’s alpha of this scale is 0.868, indicating that the scale has good reliability (Liu, 2020). In this study, the Cronbach’s alpha of this scale in stroke patients was 0.901.

#### 2.3.5 Distress thermometer (DT)

Gilson (2012) developed the Distress Thermometer for stroke patients which was subsequently translated into Chinese by Du (2018). The scale consists of a single 0 (no distress) to 10 (extreme distress) rating scale (DT) and associated Problem List (PL). Du (2018) study established a significant psychological distress cut-off score of 5. The Cronbach’s alpha of the Chinese version of the scale is 0.808, with content validity indices all >0.75, indicating good reliability and validity. In this study, the Cronbach’s alpha for the scale was measured to be 0.801.

### 2.4 Statistical analysis

IBM SPSS 25.0 and its Process v3.4 program were used for statistical analysis, with a significance level set at 0.05. Frequency and percentages are used to describe the general characteristics of stroke patients. Mean (standard deviation SD) or median and interquartile range (IQR) are used to describe the scores of coping style, resourcefulness, self-perceived burden, and psychological distress. Spearman correlation analysis was used to examine the correlation among coping style, resourcefulness, self-perceived burden and psychological distress in stroke patients. Model 6 of Process v3.4 was used for chain mediation effect analysis, utilizing 5,000 bootstrap resamples to test mediation effects, whereby a 95% confidence interval excluding zero indicates a significant mediation effect.

## 3 Results

A total of 432 stroke patients participated in the survey, with 408 valid datasets. The average age of the respondents was (64.84 ± 10.94) years, ranging from 26 to 89 years. Among them, there were 244 males (59.80%) and 164 females (40.20%). 343 cases had a spouse (84.07%); 217 (53.19%) were urban residents; education level was mainly primary school or below (49.02%); the average monthly household income was mostly between 1,001 and 3,000 Yuan (34.31%); spouses served as the primary caregivers in 51.47% of cases; among these, 386 (94.61%) were ischemic strokes, with 267 (65.44%) being first-time strokes (Table 1).

**Table 1:** General information of stroke patients (*n* = 408).

Variables	Item	Frequency (n)	Percentage (%)
Gender	Male	244	59.80
	Female	164	40.20
Age (years)	18 ~ 44	8	1.96
	45 ~ 59	143	35.05
	60 ~ 74	165	40.44
	≥75	92	22.55
Residence	Rural	191	46.81
	Urban	217	53.19
Education level	Primary school or below	200	49.02
	Junior high school	140	34.31
	Senior high school / Technical secondary school	50	12.25
	College or above	18	4.42
Occupational status	Unemployed	105	25.74
	Farmer	126	30.88
	Retired	114	27.94
	Employed	63	15.44
Marital status	Without spouse	65	15.93
	With spouse	343	84.07
Average monthly household income (Yuan)	0 ~ 1,000	108	26.47
	1,001 ~ 3,000	140	34.31
	3,001 ~ 5,000	122	29.91
	≥5,001	38	9.31
Primary caregiver	Spouse	210	51.47
	Children	170	41.67
	Others	28	6.86
Medical payment method	Self-payment	25	6.13
	Basic Medical Insurance	276	67.64
	Employee Basic Medical Insurance	107	26.23
Timely treatment within 6 Hours of onset	Yes	181	44.36
	No	227	55.64
Stroke type	Ischemic stroke	386	94.61
	Hemorrhagic stroke	12	2.94
	Mixed type stroke	10	2.45
First onset	Yes	267	65.44
	No	141	34.56
Comorbid chronic diseases	None	85	20.83
	1 type	177	43.38
	2 types	118	28.93
	3 or more types	28	6.86
Stroke duration	<6 months	286	70.10
	6 ~ 11 months	12	2.94
	1–3 years	44	10.78
	>3 years	66	16.18
ADL	Independent	55	13.48
	Mild dependence	231	56.62
	Moderate dependence	89	21.81
	Severe dependence	33	8.09

ADL, Activities of Daily Living.

The total score on the Medical Coping Mode Questionnaire was 36 (32, 39), with a confrontation dimension scores of 14 (11, 18), 12 (9, 15) for the avoidance dimension, and 8(6, 12) for the resignation dimension. The resourcefulness scale score was (69.29 ± 15.32) and the self-perceived burden scale score was (31.24 ± 7.69). The Distress Thermometer score was (4.84 ± 2.72), with 200 participants (49.02%) scoring 5 or above, suggesting a significant psychological distress detection rate of 49.02% (DT ≥ 5).

Spearman correlation analysis results showed that psychological distress was negatively correlated with resourcefulness and confrontation coping, and positively correlated with avoidance, resignation coping, and self-perceived burden (all *p* < 0.05); Additionally, self-perceived burden was negatively related to confrontation coping and resourcefulness, and positively correlated with avoidance and resignation coping (both *p* < 0.01); resourcefulness was positively correlated with confrontation coping, and negatively correlated with avoidance and resignation coping (both p < 0.01); details in Table 2.

**Table 2:** Correlation analysis of coping styles, resourcefulness, self-perceived burden, and distress (*n* = 408).

Variables	1	2	3	4	5	6
1 Confrontation	1					
2 Avoidance	−0.555^**^	1				
3 Resignation	−0.522^*^	0.428^**^	1			
4 Resourcefulness	0.676^**^	−0.417^**^	−0.593^**^	1		
5 Self-perceived burden	−0.337^**^	0.253^**^	0.624^**^	−0.391^**^	1	
6 Psychological distress	−0.554^**^	0.404^**^	0.756^**^	−0.743^**^	0.744^**^	1

^*^p < 0.05; ^**^p < 0.01.

**Figure 2:**
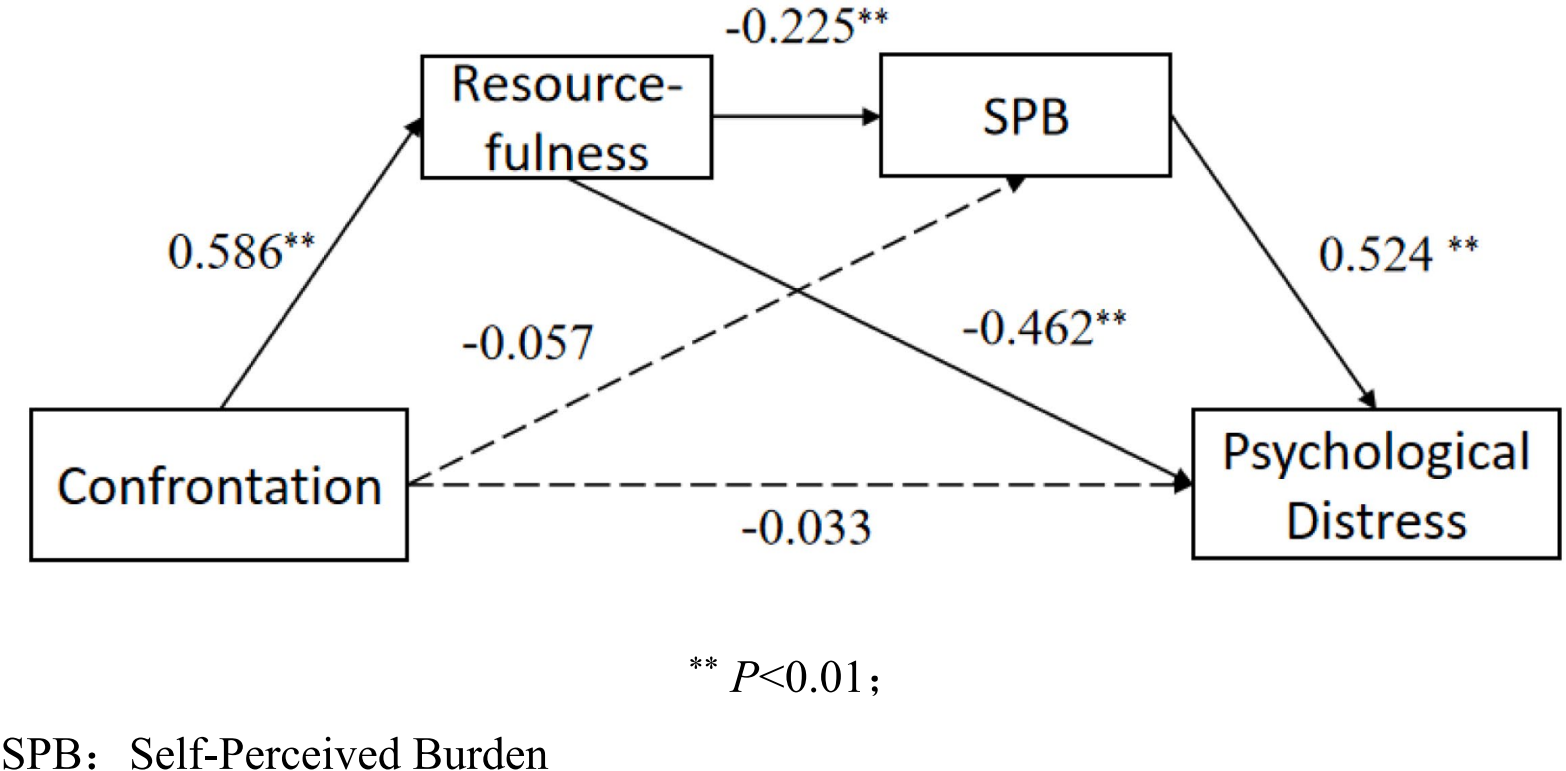
Chain mediating effect of resourcefulness, self-perceived burden on coping style (confrontation) and distress ^**^p < 0.01; ^*^p < 0.05; SPB, Self-Perceived Burden.

Using confrontation coping as the independent variable, resourcefulness and self-perceived burden as mediating variables, and psychological distress as the dependent variable, after controlling for the influence of covariates, model 6 of the Process program was used to conduct chain mediation analysis, and the Bootstrap method was used to test the mediation effect. As shown in Table 3, the total effect of confrontation on psychological distress was −0.402 (95% CI: −0.492, −0.313; *p* < 0.001), with a direct effect of −0.033 (95% CI: −0.097, 0.032; *p* = 0.282), and a total indirect effect of −0.370 (95% CI: −0.450, −0.290), accounting for 92.04% of the total effect. The chain mediation effect of resourcefulness and self-perceived burden between confrontation and psychological distress was −0.069 (95% CI: −0.111, −0.030), accounting for 17.16% of the total effect (Table 4 and Figure 2).

**Table 3:** Chain mediating effect of resourcefulness, self-perceived burden on coping style (confrontation) and distress (*n* = 408).

Variables	*R^2^*	*F*	*Coeff*	*SE*	*t*	*P*	*LICI*	*ULCI*
Outcome: resourcefulness	0.505	33.573^**^						
confrontation			0.586	0.042	13.840	<0.001	0.503	0.669
Outcome: SPB	0.238	9.475^**^						
confrontation			−0.057	0.064	−0.895	0.372	−0.183	0.069
resourcefulness			−0.225	0.063	−3.607	<0.001	−0.348	−0.103
Outcome: psychological distress	0.803	114.726^**^						
confrontation			−0.033	0.033	−0.995	0.320	−0.097	0.032
resourcefulness			−0.462	0.032	−14.292	<0.001	−0.525	−0.398
SPB			0.524	0.026	20.448	<0.001	0.474	0.574
Total Effect	0.428	24.611^**^	−0.402	0.045	−8.838	<0.001	−0.492	−0.313
Indirect effect			−0.370	0.040			−0.450	−0.290

^**^p < 0.01; SPB: self-perceived burden.

**Table 4:** Indirect effects of resourcefulness and self-perceived burden on coping style (confrontation) and distress.

Path	*Coeff*	*SE*	*LICI*	*ULCI*	Mediation effect ratio
Total indirect effect	−0.370	0.040	−0.450	−0.290	92.04%
Confrontation → resourcefulness → psyc-hological distress	−0.271	0.027	−0.326	−0.222	67.41%
Confrontation → SPB → psychological distress	−0.030	0.035	−0.097	0.038	7.46%
Confrontation → resourcefulness → SPB → psychological distress	−0.069	0.020	−0.111	−0.030	17.16%

SPB, self-perceived burden.

**Figure 3:**
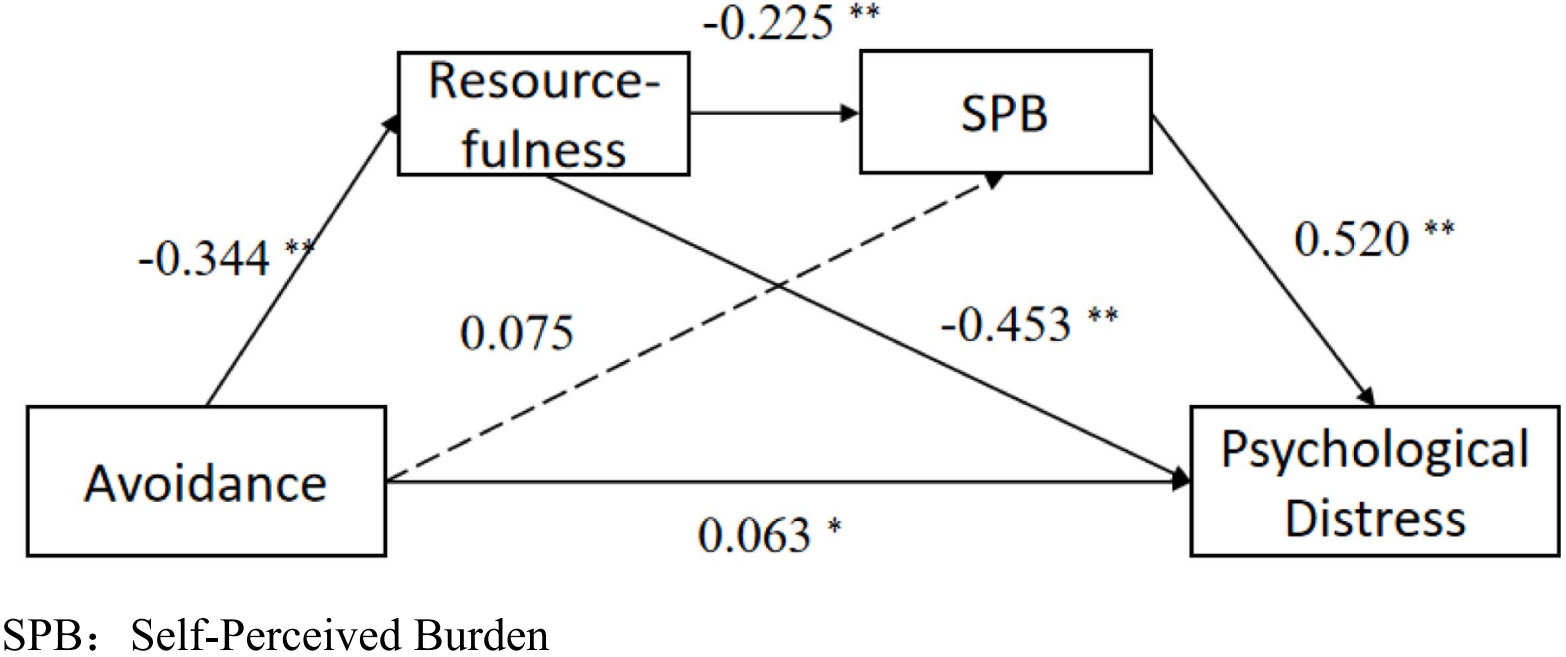
Chain mediating effect of resourcefulness, self-perceived burden on coping style (avoidance) and distress. *p < 0.05; **p < 0.01.

Using avoidance coping as the independent variable, resourcefulness and self-perceived burden as mediating variables, and psychological distress as the dependent variable, the same method was applied to examine the chain mediation effect. The model results are shown in Table 5. The results indicate that the total effect of avoidance on psychological distress was 0.299 (95% CI: 0.219, 0.378; *p* < 0.001), with a direct effect of 0.063 (95% CI: 0.014, 0.113; *p* = 0.012), and a total indirect effect of 0.235 (95% CI: 0.173, 0.302), accounting for 78.59% of the total effect. The chain mediation effect of resourcefulness and self-perceived burden between avoidance coping and psychological distress was 0.040 (95% CI: 0.020, 0.064), accounting for 13.38% of the total effect (Table 6 and Figure 3).

**Table 5:** Chain mediating effect of resourcefulness, self-perceived burden on coping style (avoidance) and distress (*n* = 408).

Variables	*R^2^*	*F*	*Coeff*	*SE*	*t*	*P*	*LICI*	*ULCI*
Outcome: resourcefulness	0.375	19.760^**^						
avoidance			−0.344	0.041	−8.348	<0.001	−0.42	−0.263
Outcome: SPB	0.241	9.628^**^						
avoidance			0.075	0.049	1.522	0.129	−0.022	0.172
resourcefulness			−0.225	0.055	−4.046	<0.001	−0.334	−0.115
Outcome: psychological distress	0.806	116.687^**^						
avoidance			0.063	0.025	2.530	0.012	0.014	0.113
resourcefulness			−0.453	0.029	−15.802	<0.001	−0.510	−0.397
SPB			0.520	0.026	20.400	<0.001	0.470	0.570
Total effect	0.398	21.732^**^	0.299	0.041	7.378	<0.001	0.219	0.378
Indirect effect			0.235	0.033			0.173	0.302

^**^p < 0.01; SPB, self-perceived burden.

**Table 6:** Indirect effects of resourcefulness and self-perceived burden on coping style (avoidance) and distress.

Path	*Coeff*	*SE*	*LICI*	*ULCI*	Mediation effect ratio
Total indirect effect	0.235	0.033	0.173	0.302	78.59%
Avoidance → resourcefulness → psychological distress	0.156	0.021	0.117	0.198	52.17%
Avoidance → SPB → psychological distress	0.039	0.028	−0.013	0.095	13.04%
Avoidance → resourcefulness → SPB → psychological distress	0.040	0.011	0.020	0.064	13.38%

SPB, self-perceived burden.

## 4 Discussion

We found that the average psychological distress score among stroke patients was (4.84 ± 2.72), with 49.02% of patients exhibiting significant psychological distress. This suggests that psychological issues are widespread among stroke patients in this survey, nearly half of stroke patients may need psychological support. Such frequent psychological distress may stem from the impact of stroke on patients’ autonomy and independence, as many must rely on others for long-term care, while also facing shifts in family and social roles and the financial burden of the disease. These factors collectively contribute to the high incidence of psychological distress. This finding is consistent with the studies of Du (2018) and Liu Z. (2023), further highlighting the need for psychological screening in the stroke population.

We found that the self-perceived burden among stroke patients, as assessed in this survey, was moderate, consistent with the findings of Wei et al. (2020) and Chen et al. (2022), but higher than the results of Zhu et al. (2023) in younger stroke patients. This may be because our survey includes more older patients who, compared with younger stroke patients, find it more difficult to cope with the physical damage and rehabilitation challenges caused by the disease, resulting in a heavier self-perceived burden. Furthermore, we also showed a positive correlation between self-perceived burden and psychological distress in stroke patients. Leroy et al. (2016) suggested that when there is an imbalance in the give-and-take between a patient and their caregiver, psychological imbalances such as guilt and self-blame may arise, contributing to self-perceived burden and becoming a source of psychological distress (such as anxiety and depression).

The coping style scores in this survey are similar to the findings of He et al. (2023) in elderly stroke patients. Data analysis showed that confrontation coping was negatively correlated with psychological distress in stroke patients, while avoidance and resignation coping were positively correlated with psychological distress. One study suggests that individuals facing stress may mitigate their experience of psychological distress to some extent through active problem-solving strategies and seeking social support (Rong et al., 2022). Other studies show that patients who adopt avoidance coping tend to avoid discussing their illness (Newcomb et al., 2024). while those who use resignation coping are more likely to withdraw from social activities, which may negatively affecting their emotional and social functioning (Jiang, 2021), these coping strategies are all potentially associated with higher levels of psychological distress (Wang et al., 2020). Based on these findings, focusing on patients’ coping styles in clinical practice and providing psychological guidance to those using negative coping mechanisms may help improve their psychological well-being.

In this survey, the resourcefulness score of stroke patients was 69.29 ± 15.32, lower than the results from Zhu et al.’s study on younger stroke patients (Zhu, 2020). This may be because the sample in this study mainly consisted of older patients, whose physical and cognitive abilities have declined, making them less capable of handling tasks independently compared to younger patients. Zauszniewski et al. (2007) emphasized the importance of resourcefulness in the health of older adults. There was a negative correlation between resourcefulness and psychological distress, consistent with previous research findings (Zhu et al., 2023; Wang et al., 2023). There is evidence that personal resourcefulness helps cancer patients reduce negative thoughts and depressive symptoms (Huang et al., 2010), while social resourcefulness aids individuals in better understanding and using available resources, thereby reducing psychological burden (Zauszniewski et al., 2022).

The results indicate that both confrontation and avoidance coping can indirectly influence psychological distress through resourcefulness and self-perceived burden. Specifically, confrontation coping positively affects resourcefulness, while avoidance coping negatively impacts it. Furthermore, resourcefulness is associated with a reduction in self-perceived burden, which ultimately affects psychological distress. These results partially support the research hypothesis. The reason for this can be attributed to the fact that confrontation coping helps stroke patients face the reality of their illness, facilitating proactive treatment and rehabilitation. This approach also encourages patients to seek family and social support, which contributes to the enhancement of resourcefulness. In contrast, avoidance coping may limit the development of resourcefulness. Further, resourcefulness is associated with an individual’s cognitive and emotional regulation abilities (Zhang, 2023). A higher level of resourcefulness may enhance one’s ability to cope with stress and regulate emotions, as well as promote better use of personal and social resources. These factors may collectively contribute to a reduction in self-perceived burden, thereby alleviating psychological distress. Conversely, patients with lower levels of resourcefulness may have fewer coping resources and struggle to manage stress effectively. For stroke patients, a lack of effective coping mechanisms can also negatively impact rehabilitation outcomes (Zhang et al., 2023), thereby increasing their self-perceived burden and psychological distress. Based on the above findings, active coping may create a mutually reinforcing relationship with resourcefulness, a mechanism that may help alleviate patients’ self-perceived burden and psychological distress.

In summary, our preliminary study revealed the potential pathways through which confrontation and avoidance coping affect the psychological distress of stroke patients through resourcefulness and self-perceived burden, providing a new perspective for understanding the relationship between the four, though further longitudinal research is needed to validate these findings. Compared with previous studies that focused on a single causal path, this study attempts to integrates resourcefulness theory with psychological stress theory to explore the potential pathway among coping style, resourcefulness, self-perceived burden, and psychological distress. The findings suggest that clinical healthcare professionals should implement interventions from multiple perspectives, jointly participate in intervention measures with caregivers, cultivate patients’ comprehensive coping ability while providing emotional support to reduce their current psychological distress and self-perceived burden. Additionally, effective coping mechanism is beneficial for patients to effectively deal with challenges and pressures in the long-term rehabilitation process and promote their rehabilitation process. However, the effectiveness of these interventions in clinical practice and continuous nursing needs to be validated through more rigorous measures, with the goal of providing more scientifically based psychological support for stroke patients.

## 5 Limitations

This study still has certain limitations. First, the sample was drawn from hospitalized stroke patients in a specific region, which may limit the representativeness of the findings and their generalizability to patients in home or community rehabilitation settings. Future research could involve multi-center studies to enhance the scientific rigor and applicability of the conclusions. Second, the cross-sectional design of the study cannot reveal causal relationships between variables. In the future, longitudinal or comprehensive studies could provide insights into the dynamic development of coping styles, resourcefulness, self-perceived burden, and psychological distress in stroke patients, and explore causal relationships. Finally, the study used self-reported questionnaires, which may introduce subjectivity, and did not conduct a multi-dimensional, in-depth analysis of psychological distress. This may have overlooked specific psychological issues faced by patients. These limitations restrict the generalizability and applicability of the findings, so the results should be interpreted with caution.

## 6 Conclusion

We found that the coping style of stroke patients can affect psychological distress through the mediating effects of resourcefulness and self-perceived burden resourcefulness and self-perceived burden play a chain-mediated role between coping styles (confrontation, avoidance) and psychological distress. The findings contribute to expanding the understanding of resourcefulness theory and stress theory, suggesting that plans can be formulated from the perspective of coping styles, resourcefulness, and self-perceived burden. Caregivers may play a role in these interventions, helping to enhance patients’ coping mechanisms and emotional support. This approach may assist in alleviating patients’ psychological distress and self-perceived burden, potentially improving the effectiveness and sustainability of clinical psychological care.

## Data Availability

The raw data supporting the conclusions of this article will be made available by the authors, without undue reservation.
