# Supplementary material for: Chain mediation of resourcefulness and self-perceived burden between coping styles and psychological distress in stroke patients: a cross-sectional study
Source: Front Psychol. 2025 Sep 5;16:1560348. doi: 10.3389/fpsyg.2025.1560348 (PMC12446327; doi:10.3389/fpsyg.2025.1560348)
Supplement: Supplementary file 1 [file Table_1.pdf]

Supplementary material

Table 1.The Strengthening the Reporting of Observational studies in Epidemiology (STROBE) statement Checklist of Items

	ITEM	Recommendation	PAGE
**Title and abstract**	1	(a)Indicate the study's design with a commonly used term in the title or the abstract	Title and abstract
		(b)Provide in the abstract an informative and balanced summary of what was done and what was found	Abstract
**Introduction**			
Background/rationale	2	Explain the scientific background and rationale for the investigation being reported	Introduction
Objectives	3	State specific objectives, including any prespecified hypotheses	Introduction
**Methods**			
Study design	4	Present key elements of study design early in the manuscript	Study Design and Participants
Setting	5	Describe the setting, locations, and relevant dates, including periods of recruitment, exposure, follow-up, and data collection	Materials and Methods
Participants	6	Give the eligibility criteria, and the sources and methods of selection of participants	Procedures
Variables	7	Clearly define all outcomes, exposures, predictors, potential confounders, and effect	Measurement

	ITEM	Recommendation	PAGE
		modifiers; give diagnostic criteria, if applicable	
Data sources/measurement	8*	For each variable of interest, give sources of data and details of methods of assessment (measurement); describe comparability of assessment methods if there is more than one group	Measurement
Bias	9	Describe any efforts to address potential sources of bias	Procedures
Study size	10	Explain how the study size was arrived at	Study Design and Participants
Quantitative variables	11	Explain how quantitative variables were handled in the analyses; if applicable, describe which groupings were chosen and why	Statistical Analysis
tatistical methods	12	(a) Describe all statistical methods, including those used to control for confounding	Statistical Analysis
		(b) Describe any methods used to examine subgroups and interactions	Statistical Analysis
		(c) Explain how missing data were addressed	N/A
		(d) If applicable, describe analytical methods taking account of sampling strategy	Measurement
		(e) Describe any sensitivity analyses	N/A
**Results**			
Participants	13*	(a) Report numbers of individuals at each stage of study—eg numbers potentially	Results

	ITEM	Recommendation	PAGE
		eligible, examined for eligibility, confirmed eligible, included in the study, completing follow-up, and analysed	
		(b) Give reasons for non-participation at each stage	N/A
		(c) Consider use of a flow diagram	N/A
Descriptive data	14*	(a)Give characteristics of study participants (e.g., demographic, clinical, social) and information on exposures and potential confounders	Results
		(b)Indicate number of participants with missing data for each variable of interest	N/A
Outcome data	15*	Report numbers of outcome events or summary measures	Results
Main results	16	(a)Give unadjusted estimates and, if applicable, confounder-adjusted estimates and their precision (eg, 95% confidence interval). Make clear which confounders were adjusted for and why they were included	Results
		(b) Report category boundaries when continuous variables were categorized	Table1
		(c)If relevant, consider translating estimates of relative risk into absolute risk for a meaningful time period	N/A
Other analyses	17	Report other analyses done - e.g., analyses of subgroups and interactions, and sensitivity analyses	Results

	ITEM	Recommendation	PAGE
**Discussion**			
Key results	18	Summarize key results with reference to study objectives	Discussion
Limitations	19	Discuss limitations of the study, taking into account sources of potential bias or imprecision; discuss both direction and magnitude of any potential bias	Limitations
Interpretation	20	Give a cautious overall interpretation of results considering objectives, limitations, multiplicity of analyses, results from similar studies, and other relevant evidence	Discussion
Generalizability	21	Discuss the generalizability (external validity) of the study results	Discussion
**Other information**			
Funding	22	Give the source of funding and the role of the funders for the present study and, if applicable, for the original study on which the present article is based	N/A
